# A comprehensive dataset of annotated brain metastasis MR images with clinical and radiomic data

**DOI:** 10.1038/s41597-023-02123-0

**Published:** 2023-04-14

**Authors:** Beatriz Ocaña-Tienda, Julián Pérez-Beteta, José D. Villanueva-García, José A. Romero-Rosales, David Molina-García, Yannick Suter, Beatriz Asenjo, David Albillo, Ana Ortiz de Mendivil, Luis A. Pérez-Romasanta, Elisabet González-Del Portillo, Manuel Llorente, Natalia Carballo, Fátima Nagib-Raya, Maria Vidal-Denis, Belén Luque, Mauricio Reyes, Estanislao Arana, Víctor M. Pérez-García

**Affiliations:** 1 Mathematical Oncology Laboratory (MOLAB), University of Castilla-La Mancha, Ciudad Real, Spain; 2 Medical Image Analysis Group, ARTORG Research Center, Bern, Switzerland; 3 Radiology Department, Hospital Regional Universitario de Málaga, Málaga, Spain; 4 Radiology Department, MD Anderson Cancer Center, Madrid, Spain; 5 Radiology Department, Sanchinarro University Hospital, HM Hospitales, Madrid, Spain; 6 Radiation Oncology Department, Hospital Universitario de Salamanca, Salamanca, Spain; 7 Radiology Department, Fundación Instituto Valenciano de Oncología, Valencia, Spain

**Keywords:** Metastasis, Translational research, Applied mathematics

## Abstract

Brain metastasis (BM) is one of the main complications of many cancers, and the most frequent malignancy of the central nervous system. Imaging studies of BMs are routinely used for diagnosis of disease, treatment planning and follow-up. Artificial Intelligence (AI) has great potential to provide automated tools to assist in the management of disease. However, AI methods require large datasets for training and validation, and to date there have been just one publicly available imaging dataset of 156 BMs. This paper publishes 637 high-resolution imaging studies of 75 patients harboring 260 BM lesions, and their respective clinical data. It also includes semi-automatic segmentations of 593 BMs, including pre- and post-treatment T1-weighted cases, and a set of morphological and radiomic features for the cases segmented. This data-sharing initiative is expected to enable research into and performance evaluation of automatic BM detection, lesion segmentation, disease status evaluation and treatment planning methods for BMs, as well as the development and validation of predictive and prognostic tools with clinical applicability.

## Background & Summary

Brain metastases (BMs) represent the most common intracranial neoplasm in adults. They affect around 20% of all cancer patients^1–6^, and are among the main complications of lung, breast and colorectal cancers, melanoma or renal cell carcinomas^1–4^. The increasing availability of systemic treatments has improved the prognosis of patients with primary tumors, leading to an increase in the probability of developing BMs^2,3,6,7^.

BMs often appear as multiple lesions, with only around 25% of patients harboring a single BM^2,8^. On magnetic resonance imaging (MRI) studies, they are found to present contrast-enhancing features. Contrast-enhanced T1-weighted (CE-T1-W) MRI is the gold standard imaging sequence for BMs, providing information about lesion size, morphology and surrounding healthy structures^7,9^. T2-weighted imaging and fluid attenuation inversion recovery (FLAIR) MRI sequences are also used to help in identifying BMs, due to the surrounding edema found in many BM lesions^1,5,7^.

Treatment of BMs typically includes a combination of radiotherapy, chemotherapy, immunotherapy, targeted therapies, and/or surgery^1–3^. Radiotherapy schemes include whole brain radiation therapy and stereotactic radiosurgery (SRS). SRS is considered the standard of care in patients with limited metastatic burden^6,7,9–11^.

The clinical management of BMs undergoing radiotherapy requires time-consuming processes such as lesion identification and segmentation^2,3,12^. Time spent on those tasks could be reduced with the aid of semi-automatic or automatic computer-guided algorithms. Machine learning (ML) and deep learning (DL) techniques are being developed for different problems related to BMs, such as: automatic BM detection^5–7,12–14^, segmentation^11,13–15^ and differential diagnosis of BMs from other brain tumors^7,12,16^. AI algorithms may also reduce human errors in all of those jobs that result from heavy workloads, allowing for increased reproducibility^6,12^.

Another problem in which AI can be helpful is the differentiation between post-treatment BM progression and radiation necrosis, a transient inflammatory effect after SRS. These two situations have overlapping features on MRI sequences, which makes it challenging to distinguish them visually^7,9,10^. Incorrect classification leads to unnecessary treatments and substantial patient harm. For this reason, AI methods have have been developed to automatically distinguish them^7,9^.

Finally, the development of prognostic and predictive metrics using the information contained in medical images is of the utmost importance because of the clinical implications. For BMs, the Graded Prognostic Assessment (GPA) index is the most popular clinically-validated prognostic scale^1,3^. However, it does not use any imaging information, but only clinical variables. In this sense, the field of Radiomics has the potential to improve the prognostic and predictive value of GPA and set the ground for novel indexes^17,18^. Radiomic-based research in brain tumors has been huge, and a variety of parameters have been studied^4,7,16,19–22^. Additionally, while morphological features obtained from MRI have proven effective in the setting of other brain tumors, little research has been done on their utility for BMs.^23–29^. The calculation of those biomarkers relies on brain tumor segmentations. Several approaches constructed using ML and DL algorithms have been proposed in the literature to automate this procedure^11,12,30–34^. However, due to the lack of large BM public datasets, there is no common ground on which they can be properly compared.

Publicly available datasets of BMs are limited. The most popular repository of images for cancer research is The Cancer Imaging Archive (TCIA)^35^, including more than 140 imaging repositories of different human cancers. However, in the case of BMs, only one database including 156 whole brain MRI studies have been found available^14^. This leads to the fact that while there is a good amount of public data for the much less frequent primary brain tumors such as glioblastoma, available datasets for BMs are scarce.

This study tries to solve that problem by contributing longitudinal magnetic resonance imaging studies of 75 BM patients, harboring 260 BM lesions, for a total of 637 imaging studies. Imaging studies include pretreatment post-contrast T1-w sequences, and most of them include other sequences such as T1, T2, FLAIR, DWI, etc. Semi-automatic segmentations of 154 different BMs for a total of 593 post-contrast T1-W segmentations are also provided with the dataset. These data are accompanied by an extensive database including clinical data and a set of morphological and radiomic-based features obtained from the segmentations.

MRI studies in our dataset have four times the number of segmentations than those currently publicly available^14^. Additionally, we make public three excel files, one of which contains clinical data, including patient information, details about the primary tumor, details about treatments, and the date of the patient’s death, as opposed to the already published one, which only contains information about the histology of the primary tumor.

## Methods

### Subject characteristics

Data collected include the follow-up imaging studies and clinical data of 75 BM patients from 5 different medical institutions. Inclusion criteria was defined as: deceased adult patients with pathologically confirmed diagnosis of BM between January 1, 2005 and December 31, 2021, availability of imaging studies with at least the post-contrast T1-w high-resolution sequence (pixel spacing ≤2 mm., slice thickness ≤2 mm., no gap between slices), no noise or artifacts in the images, and availability of basic clinical data (age at diagnosis, sex, treatment schemes followed, survival, etc.). Primary tumors were: Non-small cell lung cancer (NSCLC) (n = 38), small cell lung cancer (SCLC) (n = 5), breast cancer (n = 22), melanoma (n = 6), ovarian cancer (n = 2), kidney cancer (n = 1) and uterine cancer (n = 1).

The 75 patients included had a total of 260 BMs with a total of 637 imaging studies. Of those, 593 studies were semi-automatically segmented as described below.

### Image acquisition

All post-contrast T1-W sequences were obtained after intravenous administration of a single dose of contrast. The 593 imaging sequences segmented were acquired with a 1-T (n = 8), 1.5-T (n = 550) or 3.0-T (n = 35) MR imaging scanners. Regarding the MR imaging  vendors, General Electric (n = 225), Philips (n = 197), and Siemens (n = 171) medical systems were used. Other image parameters are described in Table 1.

**Table 1:** Image parameters from the 593 post-contrast T1-W images segmentations.

	Median (min-max)
Echo time (msec)	4.76 (1.51–16.74)
Repetition time (msec)	11.00 (5.74–600.00)
Pixel Spacing (mm)	0.5 (0.39–1.01)
Spacing between slices (mm)	1.00 (0.49–2.00)
Slice Thickness (mm)	1.30 (0.90–4.00)

### Segmentation procedure

**Fig. 1:**
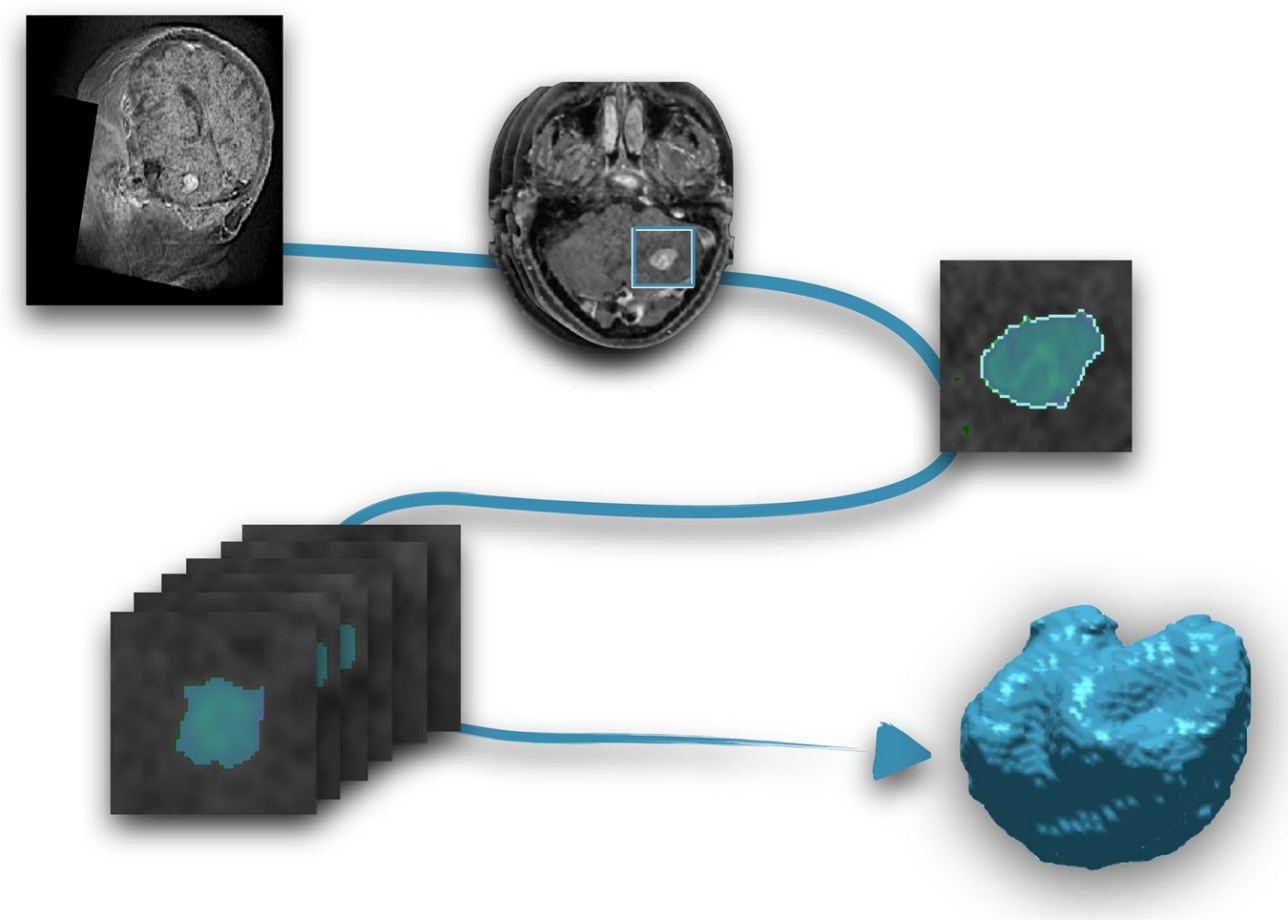
Image segmentation procedure. From the MR images (T1-W with contrast), each slice was semi-automatically segmented and manually corrected. Once every slice was segmented, the last step was the three-dimensional reconstruction of the tumor.

Segmentation was performed using an in-house semi-automatic segmentation procedure^26,28^. Tumors were automatically delineated by using a gray-level threshold chosen to identify the largest contrast-enhancing tumoral volume. Then, a biomedical engineer/applied mathematician (B. O.-T.) carefully corrected each segmentation, slice by slice, using a brushing/pixel-removing tool. The segmentation process is summarized in Fig. 1. The outcome was cross-checked by three researchers with more than seven years of expertise on MRI (D. M.-G., J. P.-B., V. M. P.-G.) and then corrected by one of the radiologists participating in the study (B.A, A.O.M, D.A, L.A.P.-R., E.A.). The raw medical images in DICOM format were used in this procedure, so they were not modified to perform the tumor segmentations.

### Clinical data and anonymization

Clinical data were collected for the 75 patients. For each patient, age at diagnosis and sex, primary tumor type and subtype, molecular markers (e.g. EGFR, ALK and ROS1 for lung cancer) and tumor stage were taken. Also, the GPA index^1,3^, was included for a subset of institutions. Regarding each BM, the ID (a number to differentiate it from other BMs in the same patient), location in the brain (frontal, temporal, parietal and occipital, right and left side), date of appearance on MRI, and treatments received were recorded. For each treatment, the type of treatment, doses, fractions, date of start and date of end were recorded. The dates of follow-up MRI studies available were also included. Radionecrosis was confirmed for 39 lesions.

The first step of the data anonymization was performed at the institutions of origin of the data. Such a step included patient and center data anonymization. An additional more profound anonymization was performed using the clinical trials processor from the medical imaging resource center^36^. Within that step, all private DICOM tags and all tags containing sensitive or identifying information as well as all dates were modified such that for every subject, the imaging study where the first BM was initially identified corresponds to January 1st, 1900. The anonymized times were computed taking as reference that time point, in days, which means that negative numbers identified treatments prior to the diagnosis of the BM. The relative differences in times for the different events for each patient were preserved. The last anonymization step was a defacing process that made impossible the facial reconstruction. After this whole process, patient records were finally reviewed independently by three of the authors (B. O.-T., J. P.-B., and J. A. R.-R.).

### Morphological parameters

Different morphological parameters were computed from the segmentations and gathered in the database, including the following:

#### Volumes

For each focus, three different types of volumes were computed: the contrast-enhancing (*V*_*CE*_), necrotic (or non-enhancing) (*V*_*N*_) and total volume (*V* = *V*_*CE*_ + *V*_*N*_).

#### Contrast-enhancing spherical rim width (CE rim width)

Obtained for each focus from the CE and necrotic volumes as

$${\rm{C}}{\rm{E}}\,{\rm{r}}{\rm{i}}{\rm{m}}\,{\rm{w}}{\rm{i}}{\rm{d}}{\rm{t}}{\rm{h}}={}^{3}\sqrt{\left(\frac{3({V}_{CE}+{V}_{N})}{4\pi }\right)}-{}^{3}\sqrt{\left(\frac{3{V}_{N}}{4\pi }\right)}.$$

By assuming that the areas of necrotic tissue and the entire tumor are spherical, this feature calculates the average width of the CE areas. Additional information and illustrations of tumors with high and low CE rim widths, can be found in^29^.

#### Surface

Obtained by reconstructing the tumor surface using the Matlab “isosurface” command from the discrete sets of voxels characterizing the tumor.

#### Surface regularity

It is a dimensionless ratio between the volume of the segmented tumor divided by the volume of a spherical tumor with the same surface. For each focus, it was calculated as

$${\rm{S}}{\rm{u}}{\rm{r}}{\rm{f}}{\rm{a}}{\rm{c}}{\rm{e}}\,{\rm{r}}{\rm{e}}{\rm{g}}{\rm{u}}{\rm{l}}{\rm{a}}{\rm{r}}{\rm{i}}{\rm{t}}{\rm{y}}=6\sqrt{\pi }\frac{{\rm{T}}{\rm{o}}{\rm{t}}{\rm{a}}{\rm{l}}\,{\rm{V}}{\rm{o}}{\rm{l}}{\rm{u}}{\rm{m}}{\rm{e}}}{\sqrt{{({\rm{T}}{\rm{o}}{\rm{t}}{\rm{a}}{\rm{l}}{\rm{s}}{\rm{u}}{\rm{r}}{\rm{f}}{\rm{a}}{\rm{c}}{\rm{e}})}^{3}}}.$$

The range for this parameter is 0 (for tumors with highly uneven surfaces) and 1 (for spherical tumors). Additional information and illustrations of tumors with high and low CE rim widths, can be found in^17^.

#### Maximum diameter

It provides the largest longitudinal measure of the tumor and is computed for each focus as the maximum distance between two points located on the surface of the CE tumor.

### Radiomic-based features

A total of 110 different features were extracted with the open-source Python package PyRadiomics version 2.2.0^37^. This feature dataset includes 16 shape descriptors and different measures of the intensity distribution and texture within the segmentation labels. The intensity features include simple first-order statistics (19 features), those derived from the gray-level co-occurrence matrix (GLCM, 24 features), gray-level run-length matrix (GLRLM, 16 features), gray-level size-zone matrix (GLSZM, 16 features), neighboring gray-tone difference matrix (NGTDM, 5 features), and gray-level dependence matrix (14 features). The features were extracted from the original image sequence after z-score normalization, intensity scaling by a factor of 100 and subsequently shifting by 300 (i. e. three standard deviations) to ensure most intensity values are positive for the first-order features and geometry tolerance 0.04. Other specific tasks may require different feature extraction procedures^18^.

No voxel resampling prior to feature extraction was used to maintain the information as unaltered as possible. Since the algorithm to extract image features is shared, any user can redo the extraction by applying any resampling.

#### Atlas location features

Affine registration was used to align all subjects to MNI atlas space^38^ using the mri_robust_register^39^. The centroid of each separate metastasis lesion was listed and may be used to efficiently identify the location and affected brain region.

### Ethical approval

We have complied with all relevant ethical regulation and all subjects included in the study are deceased. Human data were obtained in the framework of the study OpenBTAI (Open database of Brain Tumors for studies in Artificial Intelligence), a retrospective, multicenter, nonrandomized study approved by the corresponding institutional review boards: Fundación Instituto Valenciano de Oncología (2021-05), Hospital Universitario HM Sanchinarro (21.06.1858-GHM), Hospital Universitario 12 de Octubre (21/711), Hospital General Universitario de Ciudad Real (12/2021), Hospital Regional Universitario de Málaga (24/06/2021), Hospital Universitario y Politécnico La Fe (2021-504-1), MD Anderson Cancer Center (01/06/2021), Hospital Universitario de Salamanca (2021 10 879), Complejo Hospitalario Universitario de Toledo (29/9/2021-770) and Hospital Universitario Marqués de Valdecilla (14/2021 – 10/09/2021).

## Data Records

All data records collected for this manuscript are available at the Figshare Repository^40^ and on the webpage https://molab.es where the number of cases will be expanded.

Raw medical images for each follow-up study have been stored using the Digital Imaging and Communications in Medicine image file format (DICOM, ISO 12052). Tumor segmentations and the corresponding images have been stored in The Neuroimaging Informatics Technology Initiative (NIfTI) format, maintaining raw medical image coordinates, since no preprocessing was used to perform the manual segmentations. We have uploaded six zip files with the DICOMS images, one containing all the segmentations (files ended _msk.nii) and one containing the corresponding images (files ended _img.nii) to each of the segmentations available. Also, three excel files containing: (1) all the clinical data, (2) morphological parameters measured directly from the segmentations, and (3) radiomic-based features computed for each follow-up study segmented are included together with the imaging data.

## Technical Validation

### Data collection

The collaborating expert board-certified neuroradiologists identified and collected the 637 follow-up studies of the 75 BM patients included in the study. Only confirmed BM patients were included in the study, and primary tumors for each patient were pathologically confirmed and verified prior to inclusion in the study.

Data curation and testing of the inclusion criteria was performed by a biomedical engineer/applied mathematician with more than seven years experience in management of medical images (B. O.-T., D. M.-G., J. P.-B. and V. M. P.-G.) and then cross-checked by a different expert.

### Segmentation method

All semi-automatic segmentations performed in this study were carefully validated by an expert radiologist after have been performed by experienced experts in the management of medical images and cross-checked by a different expert. A reproducibility study for the methodology was performed in^26^, showing its reliability.

Each segmentation mask contains two labels for each BM: labels ending in 1 correspond to contrast-enhancing (CE) parts of the tumor; labels ending in 2 represent the non-enhancing or necrotic area of the tumor. Features were extracted for CE and necrotic zones and also were computed for the combination of both.

### Comparison between measurements obtained and radiomic features

Two excel files are provided with features from the segmented images. One of them contains some morphological variables computed directly from the manual segmentation while the other is a radiomic-based set of features.

## Usage Notes

The whole dataset can be downloaded from the figshare repository^40^. To process the provided images and segmentations, it is highly recommended that medical imaging tools be used, which handle consistently the physical space and orientation of the images. We verified that all the Nifti files (segmentations and images) can be loaded correctly with FSLeyes v1.3.0 (https://www.fsl.fmrib.ox.ac.uk) (FMRIB Centre, Oxford, UK) and DICOM files could be easily loaded using Horos v3.3.6 (https://www.horosproject.org).

## Data Availability

We provide the code used to extract the features with PyRadiomics at https://github.com/ysuter/OpenBTAI-radiomics. For reproducibility and convenience in case any user wants to customize the extraction, all the.py files needed and a “readme” file are available.
